# Protein insertion into the inner membrane of mitochondria: routes and mechanisms

**DOI:** 10.1002/2211-5463.13806

**Published:** 2024-04-25

**Authors:** Büsra Kizmaz, Annika Nutz, Annika Egeler, Johannes M. Herrmann

**Affiliations:** ^1^ Cell Biology University of Kaiserslautern, RPTU Germany

**Keywords:** carrier proteins, membrane proteins, mitochondria, protein import, TIM22 complex, TIM23 complex

## Abstract

The inner membrane of mitochondria contains hundreds of different integral membrane proteins. These proteins transport molecules into and out of the matrix, they carry out multifold catalytic reactions and they promote the biogenesis or degradation of mitochondrial constituents. Most inner membrane proteins are encoded by nuclear genes and synthesized in the cytosol from where they are imported into mitochondria by translocases in the outer and inner membrane. Three different import routes direct proteins into the inner membrane and allow them to acquire their appropriate membrane topology. First, mitochondrial import intermediates can be arrested at the level of the TIM23 inner membrane translocase by a stop‐transfer sequence to reach the inner membrane by lateral insertion. Second, proteins can be fully translocated through the TIM23 complex into the matrix from where they insert into the inner membrane in an export‐like reaction. Carriers and other polytopic membrane proteins embark on a third insertion pathway: these hydrophobic proteins employ the specialized TIM22 translocase to insert from the intermembrane space (IMS) into the inner membrane. This review article describes these three targeting routes and provides an overview of the machinery that promotes the topogenesis of mitochondrial inner membrane proteins.

## Graphical abstract

Proteins of the inner membrane of mitochondria can embark on three distinct import pathways that direct them into the inner membrane. This review describes the machinery and mechanisms by which proteins are inserted into the inner membrane by the TIM22 complex from the intermembrane space, by lateral insertion from the TIM23 complex or by conservative sorting via a matrix intermediate.

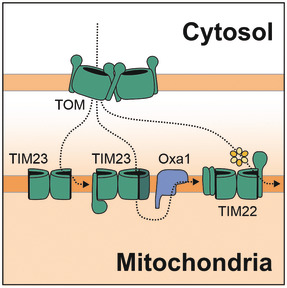

**Abbreviations**
IMS: intermembrane space
MCF: mitochondrial carrier family
MPP: mitochondrial processing peptidase
MTS: mitochondrial targeting signal
PAM: presequence translocase‐associated motor
TIM: translocase of the inner membrane of mitochondria
TOM: translocase of the outer membrane of mitochondria

## The mitochondrial inner membrane: a busy place

The two membranes of mitochondria are entirely different in nature. The outer membrane is very lipid‐rich and contains a rather low number of proteins [1]. Beta‐barrel proteins represent the most prominent class of outer membrane constituents: they form large pores to facilitate the rather unselective and rapid membrane passage of molecules [2]. Important members of the beta‐barrel family are porins (called voltage‐dependent anion channel or VDAC in humans) and Tom40, the protein‐conducting channel of the translocase of the outer membrane of mitochondria (TOM) complex. These large pores allow the diffusion of small molecules (including sugars, metabolites, amino acids, lipids, nucleotides, and ions) and, in the case of Tom40, the translocation of unfolded polypeptides. Since ions can freely diffuse through beta‐barrel proteins, there is no electrochemical gradient across the outer membrane, and the free passage of hydrogen peroxide and glutathione across the outer membrane equilibrates the redox environment of the IMS with that of the cytosol [3, 4]. In addition to the pore‐forming beta‐barrel proteins, the outer membrane contains many proteins with alpha‐helical membrane anchors which often expose large domains into the cytosol. These outer membrane proteins play important roles in the communication of mitochondria with other cellular structures; they serve as protein receptors, constituents of contact sites, mediators of apoptosis, and are even part of antiviral signaling pathways [5, 6, 7]. Regarding their biogenesis, beta‐barrel and other outer membrane proteins use specific insertion routes which were described in depth in excellent recent publications [8, 9, 10, 11, 12, 13].

With a protein‐to‐lipid ratio of about 4 : 1, the inner membrane of mitochondria is one of the most protein‐rich membranes of the eukaryotic cell [14]. It contains the oligomeric enzyme complexes of the respiratory chain and the ATP synthase which in turn form highly ordered supercomplexes [15, 16, 17]. The tight packing of enzymes into supercomplexes allows to increase the protein density of the inner membrane and to organize the membrane into cristae structures [18, 19]. Whether the supercomplex organization also supports the electron transfer between the complexes has been disputed.

Cristae are highly organized invaginations of the inner membrane that increase its surface area. The MICOS complex serves as an essential cristae organizer and constitutes oligomeric rings around the necks of the cristae thereby separating the cristae membrane from the inner boundary membrane [20, 21]. Whereas the cristae membrane predominantly houses the enzymes of the respiratory chain and the ATP synthase, the translocases of the inner membrane of mitochondria (TIM) complexes and members of the mitochondrial carrier family (MCF or carriers for short) are enriched in the inner boundary membrane [22, 23].

**Fig. 1:**
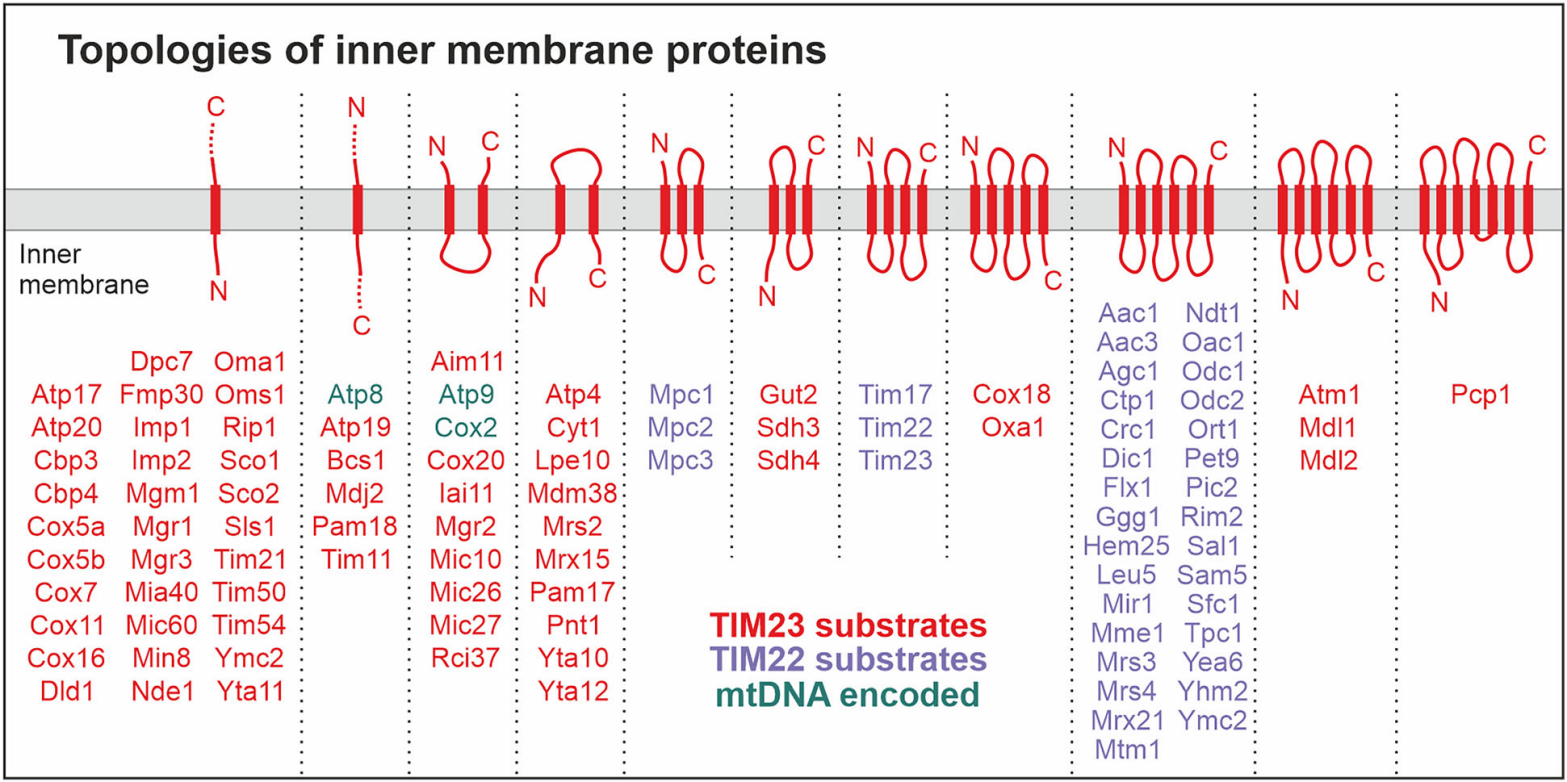
Schematic overview of the topology of inner membrane proteins. Proteins with one or several transmembrane domains are schematically represented. Shown are yeast proteins for which the topology was experimentally validated. In many cases, proteins are synthesized with an N‐terminal presequence that is proteolytically removed by MPP. Some proteins (e.g. Cyt1, Gut2) are further processed by other proteases such as the inner membrane protease Imp1/Imp2, and the topologies of the mature state of these proteins might differ. Three out of 7 mitochondrially encoded inner membrane proteins are shown in green; the proteins with more complex topology (Atp6, Cox1, Cox2 and cytochrome b) are not shown. Proteins shown in red use the TIM23 import pathway, whereas proteins in purple are inserted by the TIM22 complex.

The inner membrane contains hundreds of different proteins [24, 25, 26]. These proteins vary considerably in abundance. For example, the ATP/ADP carrier of the inner membrane (Pet9) is present in more than 180 000 copies per cell whereas some regulatory proteins, such as the translational activator Sov1, are on the other end of the spectrum and are found in only a few copies per cell [25]. Inner membrane proteins adopt very different topologies and differ in the number of transmembrane domains (Fig. 1). Most inner membrane proteins that expose their N‐termini to the matrix, are synthesized with an N‐terminal presequence encoding the matrix targeting signal (MTS). Presequences form amphipathic helices with one positively charged and one hydrophobic surface, they are rich in hydroxylated residues and lack negative charges. They are usually between 8 and 80 residues in length and can be reliably detected by prediction algorithms [27]. Presequence‐containing proteins can also reach an N‐out topology; this orientation is often seen in conservatively sorted proteins (see below).

Presequence‐containing proteins, regardless of whether they are destined for the matrix or the inner membrane, are imported by the matrix targeting (or TIM23) pathway. Carrier proteins represent another large class of inner membrane proteins and share a common structure comprising six transmembrane spans (see below). Carriers are made without presequences as their N‐termini face the IMS (N‐out topology) and they use the TIM22 translocase to be embedded into the inner membrane. The import routes of the different inner membrane proteins will be introduced in more detail in the following sections. We will thereby predominantly describe the machinery of baker's yeast from which most of our current knowledge is derived. However, the import machinery of human mitochondria is highly conserved and differs predominantly in some accessory subunits as mentioned below.

## Route 1: Stop‐transfer proteins — lateral insertion from the TIM23 translocase

The TIM23 complex is the major translocase of the inner membrane and facilitates the transport of about 60–70% of all mitochondrial proteins [25], including all proteins with N‐terminal presequences. Whereas most of the proteins fully traverse the TIM23 complex to the matrix, some membrane proteins are arrested during import and released laterally into the lipid bilayer. This mechanism is referred to as the stop‐transfer pathway and is used in particular by single‐spanning proteins of N‐in topology (Fig. 1).

### Structure of the TIM23 complex

The TIM23 translocase comprises three essential integral inner membrane proteins: Tim17, Tim23, and Tim50. Tim50 serves as an IMS‐exposed receptor that facilitates the translocation of precursors from the TOM to the TIM23 translocase [28, 29, 30]. Tim23 and Tim17 each contain four transmembrane segments and are structurally related. Initially, Tim23 was assumed to serve as the pore‐forming central subunit of the TIM23 core (hence the name TIM23 complex), however, recent structural and biochemical analyses clearly identified Tim17 as the critical protein‐conducting subunit [31, 32, 33]. The very high conservation of Tim17 and the essential nature of many of its residues (also in comparison to the less conserved Tim23 subunit) supports the critical role of Tim17 in preprotein translocation [34, 35, 36].

**Fig. 2:**
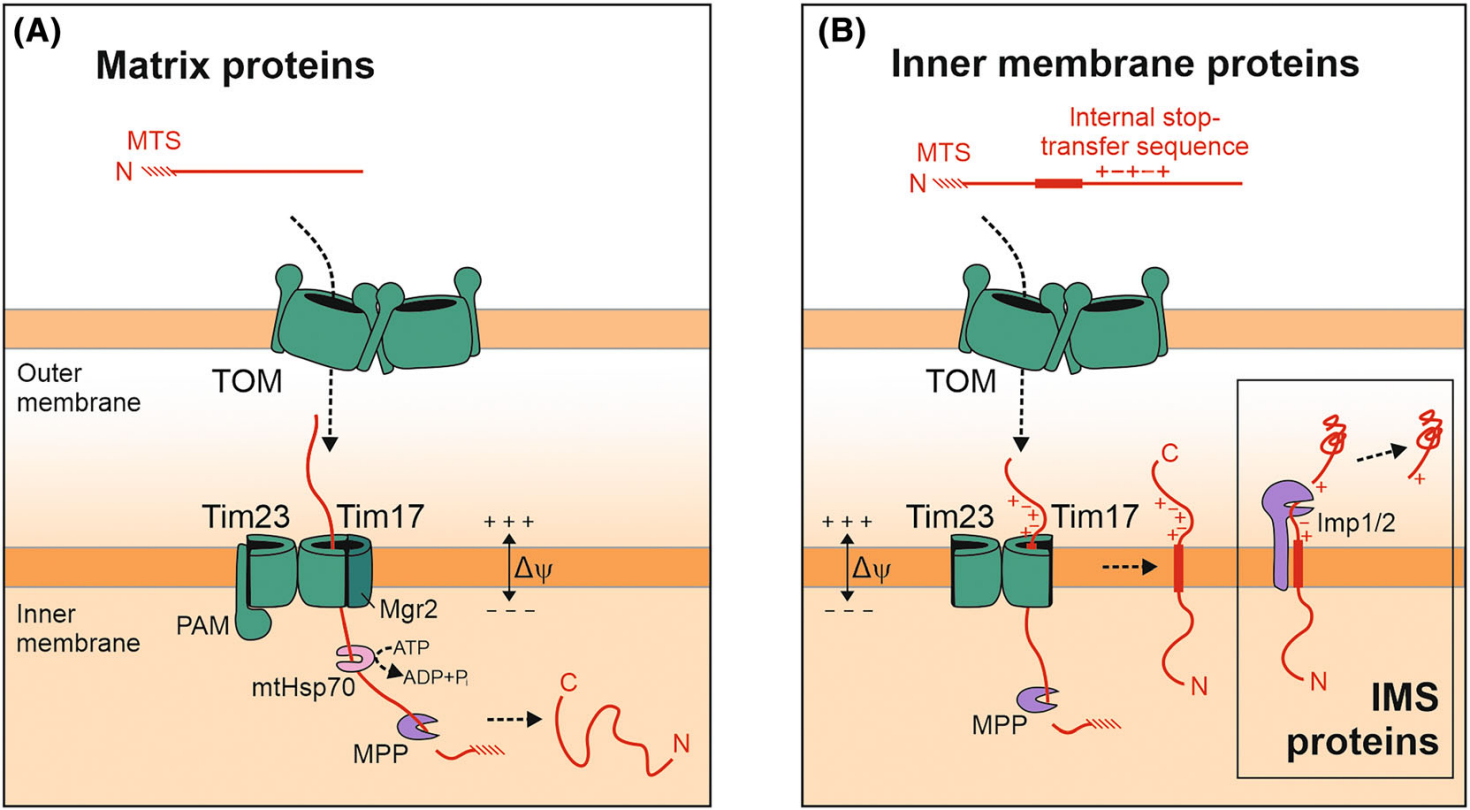
Stop‐transfer proteins are laterally released from the TIM23 complex. (A) Presequences direct proteins through the protein‐conducting channel of the TOM complex of the outer membrane. The presequences are further transferred into the matrix by the TIM23 translocase where they are proteolytically removed by MPP. (B) Stop‐transfer sequences consisting of a hydrophobic segment followed by clusters of charged residues can serve as stalling signals to arrest the translocation intermediates at the level of the inner membrane and allow their lateral insertion into the lipid bilayer. Tim17 constitutes the central component of the TIM23 complex and forms a laterally open half‐channel which, for matrix transport, can be closed by Mgr2. Translocation into the matrix is driven by the import motor (PAM complex) and the hydrolysis of ATP, whereas membrane insertion can be ATP‐independent if the stop‐transfer sequence directly follows C‐terminal to the presequence. Please note that the TIM23 complex is shown in a highly simplified schematic way, omitting several of its subunits. Inset: IMS proteins with a bipartite presequence can be released into the IMS through cleavage by the inner membrane peptidase (Imp1/Imp2 complex).

Both Tim17 and Tim23 form half‐channels that are arranged in a back‐to‐back fashion with their cavities facing away from each other (Fig. 2A). Whereas the lateral opening of Tim23 is permanently exposed to the lipid phase, the surface of Tim17 can be dynamically sealed by the non‐essential membrane protein Mgr2 (mitochondrial genome required 2). Thus, Mgr2 and Tim17 can form a channel‐like structure that is well‐suited for protein translocation [31, 32, 33]. However, upon dissociation of Mgr2, the lipid‐exposed translocation path allows the integration of inner membrane proteins directly into the lipid phase (Fig. 2B). Thus, Mgr2 serves as the lateral gatekeeper of the TIM23 translocase [37]. Additionally, Mgr2 is responsible for Tim21 recruitment to the core translocase, which in turn couples the TIM23 translocase to respiratory complexes ensuring a high local membrane potential in vicinity to the import machinery [38]. Tim23 presumably has a structural role as a platform to associate further subunits like Tim44 which recruits the ATP‐driven import motor (PAM complex) to the matrix side of TIM23.

### Protein import through the TIM23 complex

Almost all matrix proteins and most inner membrane proteins carry N‐terminal presequences to direct the proteins through the protein‐conducting channel of the TOM complex to Tim50 in the IMS. Tim50 serves as a receptor that is crucial for handing over proteins from the TOM to the membrane‐embedded Tim17 subunit. The positively charged MTS interacts with the negatively charged surface of the Tim17 cavity which drives the translocation through the inner membrane in a membrane potential‐assisted manner [32]. Despite the fact that all matrix‐destined precursors are imported by the TIM23 complex, the dependence of individual precursors on the level of the membrane potential can differ considerably and is influenced by the presequence [39] as well as by the specific properties of their mature part [30].

Within the TIM23 complex, preproteins can take two different paths (Fig. 2A,B). Matrix proteins are driven all the way across the inner membrane by mtHsp70 in an ATP‐dependent reaction [40]. The translocation of inner membrane proteins can be arrested at the level of their transmembrane domain which is laterally released from Tim17 into the lipid bilayer of the inner membrane [31, 32, 33, 41]. If the stop‐transfer sequence follows directly after the presequence, the help of the import motor is dispensable [42, 43]. The matrix processing peptidase (MPP) removes presequences from matrix and inner membrane proteins [44]. In some cases, the IMS‐exposed inner membrane protease (Imp1/Imp2) additionally cleaves stop‐transfer proteins to release their mature form into the IMS [45] (Fig. 2B, inset). Cleavage at the level of the inner membrane can also be mediated by rhomboid proteases such as Pcp1 (PARL in humans) [46, 47, 48, 49, 50, 51].

### Stop‐transfer signals in inner membrane proteins

The TIM23 complex distinguishes matrix proteins from inner membrane proteins with stop‐transfer signals that have to be arrested and laterally released. This task is not trivial, as conservatively sorted inner membrane proteins must be fully transferred into the matrix even though they often contain multiple hydrophobic transmembrane domains. Therefore, the hydrophobic segment alone does not suffice as a stop‐transfer signal. The transmembrane domains of conservatively sorted proteins are often less hydrophobic and rich in helix‐breaking proline residues [52, 53]. Furthermore, clusters of charged residues (in particular negatively charged residues) that follow the transmembrane segment C‐terminally serve as critical elements to arrest the translocation of stop‐transferred proteins in the TIM23 complex [41, 54] (Fig. 2B). These negative charges might be repelled from the negative charges in the IMS‐exposed region of Tim17 to arrest them during their import reaction [32]. Tim50 plays a crucial regulatory role and coordinates the interactions of the TIM23 complex with precursor sequences in the IMS to the recruitment of the import motor in the matrix [28, 42, 55, 56, 57]. Remarkably, within one protein, a transmembrane segment with a stop‐transfer signal can follow C‐terminally to transmembrane domains that are inserted conservatively from the matrix; such a complex organization is for example used for the topogenesis of the ABC transporter Mdl2, a protein with six transmembrane domains that are oriented in an N‐in C‐in orientation [58]. Since Mgr2 serves as a gatekeeper at the TIM23 complex, it might help to decipher the topogenic signals in inner membrane proteins [37].

### Role of Mgr2

Mrg2 was initially identified in a screen for proteins required for the survival of yeast cells without mitochondrial DNA [59]. Mgr2 is a distant member of the Tim17/Tim22/Tim23 protein family; however, it lost two of the four transmembrane segments found in the translocase subunits [36]. Mgr2 employs an unconventional import mechanism as its targeting sequence is formed by a C‐terminal extension which is removed by the inner membrane protease Imp1 following inner membrane insertion [60].

Mgr2 is a dynamic component of the TIM23 complex, but it becomes stably associated during preprotein engagement. Thereby, it seals the opening of Tim17 with its two transmembrane segments and allows efficient translocation of matrix proteins (Fig. 2A). However, once a stop‐transfer preprotein enters the Tim17 cavity, Mgr2 dissociates from the complex to facilitate the lateral release of the hydrophobic segment into the lipid bilayer [31]. Upon release of Mgr2, the cavity of Tim17 including its negatively charged patch at the entrance is exposed to lipids. This distorts the lipid bilayer and allows preprotein transport via local membrane thinning [33]. Due to this dynamic recruitment of Mgr2 to the TIM23 complex, overexpression of Mgr2 impedes the integration of transmembrane sorting signals [37]. On the contrary, in mutants lacking Mgr2, the half‐channel of Tim17 is permanently open, increasing the rate of lateral insertion.

Taken together, the TIM23 complex can be compared to a versatile two‐way valve within the machinery of mitochondrial protein import. Depending on the setting of the valve, the import flux can either be directed into the matrix or the inner membrane. In this context, Mgr2 emerges as the central gatekeeper [37], controlling the valve settings and thereby determining the fate of proteins for lateral release or matrix localization.

## Route 2: Conservative sorting — membrane insertion from the matrix via Oxa1 and Bcs1

**Fig. 3:**
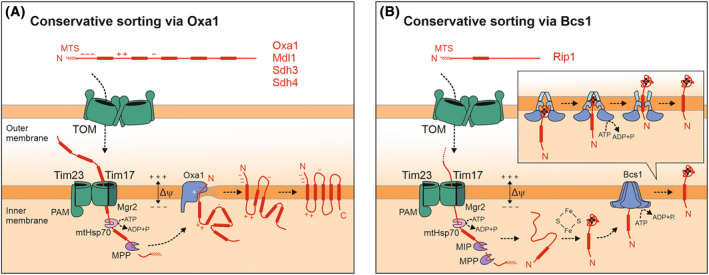
Conservative sorting of inner membrane proteins. (A) Conservatively sorted proteins reach the inner membrane in an export‐like insertion step. Initially, these proteins are translocated into the matrix where they form soluble, chaperone‐bound sorting intermediates. These intermediates then insert into the inner membrane via the assistance of the Oxa1 insertase in a reaction that depends on the membrane potential (Δψ). (B) In the case of the Rieske iron–sulfur protein Rip1, the folded domain is translocated by the Bcs1 complex across the inner membrane in an ATP‐dependent reaction. The inset depicts the individual steps of Bcs1‐mediated pushing of the folded domain across the inner membrane.

A number of inner membrane proteins are not arrested at the level of the TIM23 complex but form a matrix‐located sorting intermediate that inserts into the membrane in an export‐like fashion. This insertion route is referred to as the “conservative sorting” pathway as the mode of insertion is conserved from that of bacterial membrane proteins, which are integrated into the bacterial inner membrane [61, 62]. Import and export of the preprotein are two distinct processes that can occur independently of one another [63, 64, 65]: First, conservatively sorted proteins are driven by the TIM23 complex with the help of the import motor into the matrix (Fig. 3A,B). Following processing by MPP, these translocation intermediates are inserted into the inner membrane by the use of one of two distinct insertases: Oxa1 (cytochrome oxidase activity 1) and the Bcs1 complex. Insertases are enzymes that promote the insertion and topogenesis of membrane proteins [66].

### The Oxa1/YidC/Alb3 superfamily of protein insertases

Oxa1 was discovered as a yeast protein that is essential for the assembly of the cytochrome oxidase [67, 68, 69] and represents the founding member of the Oxa1/YidC/Alb3 superfamily of protein insertases. Members of this superfamily are found in all three kingdoms of life (archaea, bacteria, and eukaryotes) and can be grouped into two major branches. On the one hand, some homologs are derived from the bacterial YidC protein, such as Oxa1 in mitochondria and Alb3 in chloroplasts. On the other hand, there are proteins derived from the archaeal branch, which serve as core elements in several distinct insertases in the ER membrane, such as the ER membrane complex (EMC), the guided entry of tail‐anchored proteins (GET) complex and the TMCO1 insertase [66, 70, 71, 72, 73, 74].

Members of the Oxa1 superfamily share three structurally conserved transmembrane domains that form a reaction center with a hydrophilic, in most cases positively charged vestibule [75, 76]. Although their sequence identity is low, many were shown to be functionally interchangeable [77, 78, 79, 80]. The molecular structures of several Oxa1 superfamily members have recently been solved [75, 76]. Their similar architecture suggests that they all employ the same fundamental principle to catalyze membrane insertion processes which rely on two mechanisms: First, they use positively charged side chains to neutralize negative charges in their substrates during membrane translocation; and secondly, they locally distort the lipid bilayer by membrane thinning to facilitate the topogenesis of proteins [70, 72, 75, 76]. The charge preference of Oxa1 family members and the orientation of the membrane potential in bacterial, mitochondrial, and thylakoid membranes define the final topology of membrane proteins which is known as the “positive inside” rule [81, 82, 83, 84] (Fig. 3A).

### Oxa1‐mediated protein insertion into the mitochondrial inner membrane

With about 2000 copies per yeast cell, Oxa1 is a rather abundant inner membrane protein [25]. It represents the general insertion site for conservatively sorted inner membrane proteins as well as for mitochondrial encoded proteins [85, 86, 87, 88]. In order to promote the insertion of mitochondrial translation products, Oxa1 carries a C‐terminal helix that serves as a ribosome receptor [89, 90, 91]. In yeast, mitochondrial ribosomes are permanently tethered to Oxa1 in a stochiometric ratio of 1 : 1. In human mitochondria, the interaction might be less tight, but also here Oxa1 serves as a ribosome‐anchor on the inner membrane [92, 93, 94, 95]. The membrane proteins Mdm38 [96] and Mba1 (called MRPL45 in humans) serve as additional ribosome anchors on the inner membrane [92, 93, 97, 98, 99, 100].

Examples for nuclear‐encoded Oxa1 substrates that follow the conservative sorting route include Sdh3, Sdh4, Mdl1, Oxa1 itself and Atp9 in organisms in which this protein is not mitochondrially encoded. The role of Oxa1 is not restricted to the insertion of proteins into the inner membrane, but it also promotes the folding and assembly of membrane proteins [77, 88, 101, 102, 103, 104, 105, 106].

### Bcs1‐mediated protein insertion into the mitochondrial inner membrane

Bcs1 (cytochrome bc1 synthesis 1) is an integral inner mitochondrial protein that is essential for respiratory growth [107, 108]. It is a member of the AAA (ATPases associated with diverse cellular activities) family and forms a homo‐heptameric membrane‐embedded complex with a large C‐terminal region facing the matrix (Fig. 3B). This ring‐like structure forms at its center a deep, matrix‐oriented cavity that serves as active center of this inner membrane insertase. The Rieske iron–sulfur protein Rip1 is the only known substrate of the Bcs1 complex thus far. Rip1 is a central subunit of complex III of the respiratory chain. The Rip1 precursor carries a classical N‐terminal MTS which directs it into the matrix where it is cleaved off by MPP and the mitochondrial intermediate peptidase Oct1 (octapeptidyl aminopeptidase 1) [61, 109]. In the matrix, the IMS domain of Rip1 folds by insertion of an iron–sulfur cluster and formation of a disulfide bond before it is exported by the Bcs1 heptamer across the inner membrane. Translocation through the Bcs1 ring requires a substantial conformational change by which it pushes the folded C terminus through the lipid bilayer into the IMS [108, 110]. Simultaneously, a reorientation of the transmembrane helices of Bcs1 allows the lateral release of the transmembrane segment of Rip1 into the inner membrane.

## Route 3: Carrier proteins — a dedicated pathway for a special type of membrane proteins

The ATP/ADP carrier Pet9 is one of the about 35 (yeast) to 60 (human) members of the mitochondrial carrier family (MCF or SLC25), which are diverse in their abundance as well as their function. [111, 112]. Due to the less permeable nature of the inner mitochondrial membrane, mitochondrial carriers play a crucial role in transporting nucleotides, metabolites, amino acids, phosphate, iron, and many other small molecules to and from the mitochondrial matrix. Most carriers function as antiporters (such as Pet9 which exchanges ATP for ADP), but some are symporters or uniporters [111].

Structurally, carrier proteins are composed of six alpha‐helical transmembrane domains, with their N and C termini exposed to the IMS. Most carriers have a mass of about 30–34 kDa and are formed by three repetitive pairs of transmembrane domains. Each of these carrier modules exposes a positively charged loop into the matrix that is part of the internal targeting signal of carriers. Carriers lack an N‐terminal MTS and are usually not processed by mitochondrial proteases. The targeting information of mitochondrial carriers is contained in one or multiple internal targeting sequences [113, 114].

The carrier import pathway is not exclusively used by carriers but also by members of the Tim17 protein family and mitochondrial pyruvate carriers [115]. Members of the Tim17 protein family contain four transmembrane domains in an N‐out‐C‐out topology, whereas the pyruvate carriers Mpc2 and Mpc3 contain three membrane‐spanning domains and adopt an N‐out topology [116, 117, 118] (Fig. 1). Thus, proteins that use the carrier pathway consistently share an N‐out topology, use internal targeting sequences that are part of their matrix‐exposed loops and are not processed by MPP.

### Protein import by the carrier pathway

**Fig. 4:**
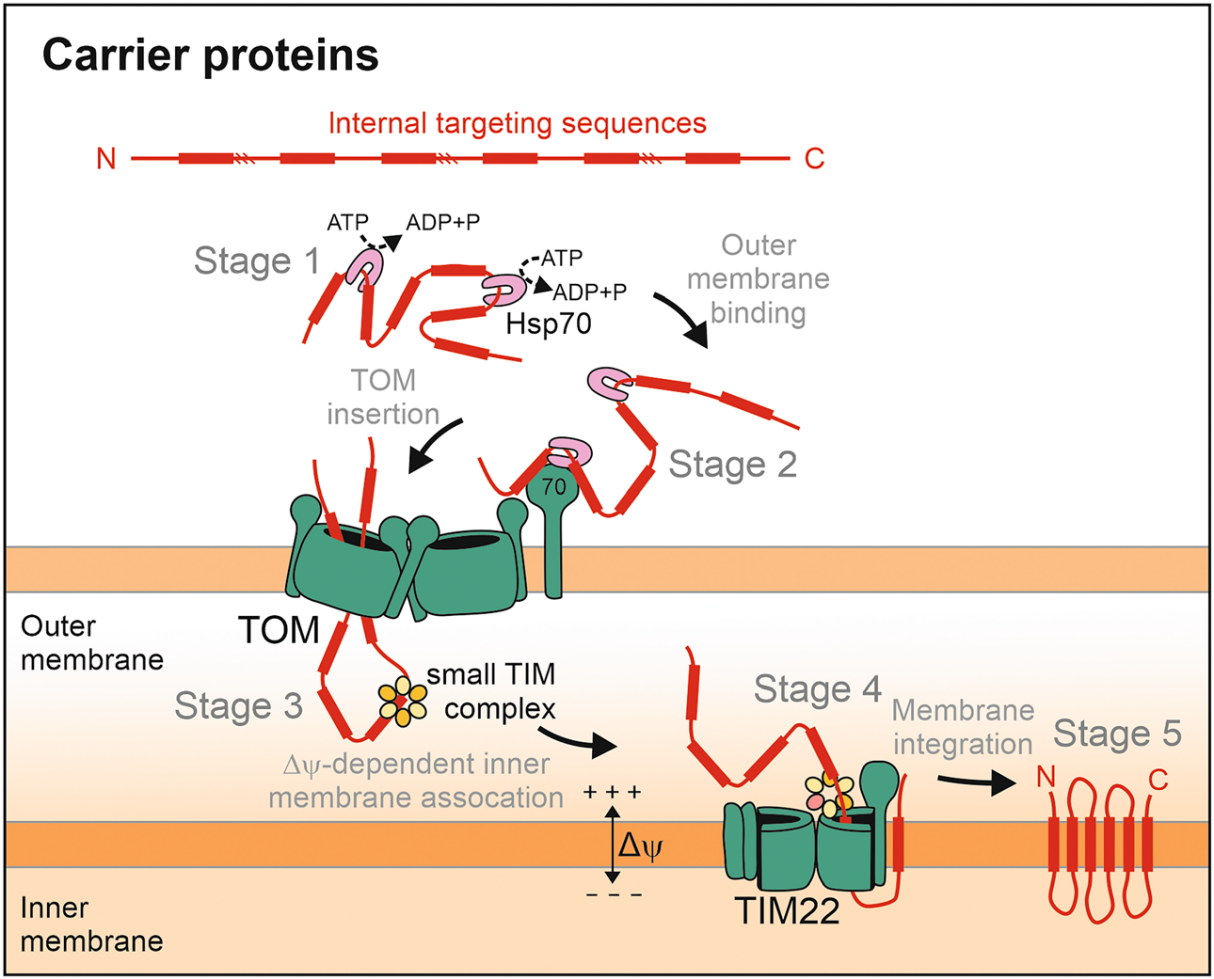
TIM22‐mediated membrane insertion of carrier proteins. Carrier proteins are bound by chaperones in the cytosol to maintain their import‐competence (stage 1) and facilitate the binding to the Tom70 receptor (stage 2). Following translocation through the TOM complex, small Tim complexes function as chaperones to facilitate the transfer of the hydrophobic carrier proteins to the TIM22 complex (stage 3). The TIM22 complex mediates the lateral insertion of carriers into the inner membrane (stage 4) where they fold into their final monomeric structure (stage 5).

Owing to their very hydrophobic nature, carrier proteins are bound by chaperones in the cytosol which keep them in an import‐competent confirmation and prevent their mistargeting to the ER [119, 120, 121]. The import of carrier proteins can be separated into five distinct stages (Fig. 4). The internal signals of the carriers are recognized by the outer membrane receptor Tom70 and threaded in a hairpin‐like conformation through the protein‐conducting pore of the TOM complex [114, 122, 123]. In the IMS, soluble hexamers of small Tim proteins (Tim9/Tim10 and Tim8/Tim13) bind to these incoming carrier loops and escort them to the TIM22 complex of the inner membrane [124, 125, 126, 127]. The ring‐like small Tim complexes use the termini of their six subunits as “tentacles” and constrict the bound carriers in a clamp‐like structure, keeping them in a nascent‐chain conformation [128, 129]. Shielding and tight holding of the carrier preprotein is realized by binding of the substrate in a highly conserved hydrophobic cleft of a hexameric ring.

Tim22 is the essential and central subunit of the TIM22 complex. Tim22 is structurally closely related to Tim17 [130], but the other subunits of the TIM22 complex (Tim54, Tim18, and Sdh3 in yeast; TIM29 and AGK (acyl glycerol kinase) in humans) have no direct counterparts in the TIM23 translocase [105, 131, 132, 133, 134]. A specialized small Tim complex is bound to the IMS‐facing side of the TIM22 complex, consisting of Tim9, Tim 10, and Tim12 [128]. This specialized chaperone complex might feed the carriers into the Tim22 subunit, which, together with the auxiliary subunits of the TIM22 complex, mediates their membrane insertion in a membrane potential‐dependent manner. The structure of Tim22 resembles that of Tim17 [130] and protein insertion might work mechanistically in an equivalent way by use of a half‐channel‐like structure that is laterally open to the lipid bilayer, however, details still have to be elucidated.

## Conclusion

Our knowledge about the insertion of inner membrane proteins is deduced from a handful of model proteins that were analyzed by *in vitro* assays with isolated yeast mitochondria. Whether the three pathways described here operate indeed independently for distinct pools of inner membrane proteins will have to be examined more comprehensively in the future. The highly similar architecture of the half‐channels formed by Tim17, Tim22, and Tim23 suggests that they all exhibit the same biochemical activity and thus, the substrate spectrum might be rather defined by the auxiliary subunits. However, the specific roles of the different subunits of the inner membrane translocases, in particular of those in the TIM22 complex, still await discovery.

## Author contributions

BK, AN, AE and JMH conceptualized the figs. BK and JMH created the images. All authors wrote the manuscript.
